# Comprehensive Preschool Screening in Upper Austria for Early Detection of Hearing Loss: Prevalence, Follow-Up, and Outcomes of the Last Eight Years

**DOI:** 10.3390/jcm15156051

**Published:** 2026-08-04

**Authors:** Veronika Moser, Julia Szegedi, Doris Detter-Biesl, Nina Rubicz, Lukas Scheuchenpflug, Paul Martin Zwittag

**Affiliations:** 1 Department of Otorhinolaryngology, Kepler University Hospital, Krankenhausstraße 9, 4020 Linz, Austria; 2 Medical Faculty, Johannes Kepler University, Linz, Altenbergerstrasse 69, 4040 Linz, Austria; 3 Speech and Language Therapy, University of Applied Sciences for Health Professions Upper Austria, 4020 Linz, Austria

**Keywords:** hearing loss, hearing screening, Speech screening, speech language pathology, articulation, grammar, hearing impairment

## Abstract

**Background/Objectives:** Children with normal newborn hearing screening results can develop hearing loss from various causes during their first years of life. Since 2016, a hearing and speech-language pathology screening has been conducted annually in kindergartens in Upper Austria in children aged 4 to 5 years. If an abnormal result is found, further evaluation is recommended. **Methods:** Data from the kindergarten hearing screening from 2018 to 2025 and hearing and speech screening from 2022/2023 were retrospectively analyzed. In addition, a prospective questionnaire survey was conducted with the parents of children who had an abnormal hearing screening result in 2024/2025. The survey asked whether the parents followed the recommendation for further evaluation and what the outcome of that evaluation was. **Results:** From 2018 to 2025, between 14,975 (2024) and 17,101 (2020) children were screened annually. The prevalence of abnormal hearing screening results ranged from 5.89% (2020) to 8.17% (2022). Abnormal grammar and articulation were found in 18.68% and 68.28% of the in-depth analyzed screenings of 4–5-year-old children in 2022, respectively. A total of 209 questionnaires were evaluated in the prospective questionnaire study. Approximately 70% of the participating parents reported having their child undergo the recommended evaluation by an ORL specialist. According to the parents, the most frequent cause of hearing impairment was middle ear effusion or Eustachian tube dysfunction. No new cases of sensorineural hearing loss were diagnosed in this population of 209 children, according to the parents surveyed. **Conclusions:** Nationwide screening in kindergartens allows for the early detection of hearing loss in early childhood, enabling children to receive evaluation and treatment. This is supported by the fact that, according to their parents, almost a quarter of the children with abnormal hearing screening results received a diagnosis requiring treatment from an ORL specialist.

## 1. Introduction

In German-speaking countries, the prevalence of hearing disorders at birth is 1.2 per 1000. To assess hearing at birth, the newborn hearing screening was introduced nationwide in Austria in 1995. The results have been recorded in the Parent–Child Health Record Book since 2003 [1]. Congenital hearing impairments can thus be detected at a very early stage, enabling optimized care for young children with hearing impairments. Hearing disorders, however, can also develop later in childhood. Often, these involve a conductive hearing disorder due to middle ear effusion. However, sensorineural hearing loss may also manifest later in childhood and may not have been present or detectable at birth. Audiological studies on school-aged children found a prevalence of sensorineural hearing loss of 2.2 per 1000 [2] or up to 7.8% for hearing loss of any cause (including conductive hearing disorders) [3]. The prevalence of middle ear effusion in early childhood is particularly high—around 80% of children entering primary school will have experienced at least one episode of middle ear effusion [4]. To ensure early detection of hearing loss in children, re-screening children’s hearing at preschool age is an essential preventative measure [5]. The program follows recommendations of the European Consensus Statement [6]. Untreated hearing impairments negatively affect language development and lead to speech delays and poorer school performance [5,7]. In Upper Austria, a comprehensive speech and hearing screening is conducted once a year by speech therapists in kindergartens using the standardized “Logik-S” screening [8]. This procedure was implemented in 2016 and includes assessing articulation, grammar, expressive and receptive language, voice, breathing, and hearing. Data from Upper Austria—the third-largest federal state in Austria and the one with the second-highest number of births [9]—on the comprehensive speech and hearing screening at preschool age has not yet been systematically analyzed. The aim of the retrospective component of this study was to create a comprehensive picture of childhood hearing impairment in Upper Austria. Comparing the results with findings from other Austrian regions as well as international studies allows for a well-founded interpretation of the data. The aim of the prospective part of the study was to ascertain the degree to which the screening recommendations resulted in diagnoses and treatments. The findings were intended to contribute to the further development and standardization of hearing screening programs.

## 2. Materials and Methods

In this monocentric retrospective study, data were included from all children who were examined by the standardized speech and hearing screening in Upper Austrian kindergartens from 2018 to 2025. This screening is conducted for every child at age four to five years as a public service.

The screening was conducted by speech therapists using the standardized screening method “Screening (LogiK-S)” [8] to test expressive and receptive language, grammar, voice, breathing (mouth/nasal breathing), and hearing. In this study, the analyses were focused on hearing, articulation, and grammar.

Hearing was assessed using screening audiometers (Oscilla SM920-P (Inmedico, Lystrup, Denmark), Maico MA 25 and Maico EasyTone(MAICO Diagnostics, Berlin, Germany), Interacoustics AS608 (Interacoustics, Middelfart, Denmark), Vero Flex Audiometer (PATH medical, Inning am Ammersee, Germany) at frequencies of 500 Hz, 1000 Hz, 2000 Hz, and 4000 Hz in both ears. Calibration of the audiometers was performed annually in accordance with the ISO 389 standard [10]. The hearing test was administered in a designated room to ensure optimal quietness, although it was not conducted in an audiometric booth. The examiners were certified speech therapists who followed standards in accordance with their training and academic backgrounds. A hearing threshold (HT) of 30 dB or above at each frequency was considered pathological. In addition to the 4 principal frequencies, the questionnaire included an overall assessment of the hearing screening (HS): normal and abnormal. The study only included children who had not been diagnosed with hearing disorders in newborn screening.

Articulation and grammar were classified as normal or abnormal. Demographic data include age at the time of screening, gender, native language, and district of kindergarten attendance.

Data from the hearing screening over the whole period 2018 to 2025 based on HS were analyzed separately.

A detailed evaluation of screening 2022/2023 was conducted based on the parameters of HS (normal, abnormal), HT (normal, abnormal, unclear), multilingualism, grammar, and articulation. First, the HT findings were classified as “normal” in case of all values of HT < 30 dB, “abnormal” in case of at least one value > 30 dB, and “unclear” in case of no HT > 30 dB and at least one missing value [11]. In cases of incoherent screening reports between HT and HS, the dataset was excluded (Appendix A Table A1).

For pairwise analyses of binary encoded variables, associations were quantified using phi coefficients along with their *p*-values (two-sided), calculated with scipy.stats.pearsonr (SciPy version 1.16.1). The assumptions required by this test, which are non-sparse contingency tables and independent observations, were considered fulfilled [12]). Missing values were excluded pairwise prior to analysis. The number of observations excluded across the analyses ranged from 146 to 285, depending on the variables that were compared. Since the analysis was exploratory, *p*-values were not adjusted for multiple comparisons. The results should therefore be interpreted with appropriate caution.

Each year, parents of children with an abnormal hearing screening result are advised by the speech-language therapist to consult an ORL specialist in order to initiate further diagnostic evaluation and, if necessary, appropriate therapeutic interventions. A prospective questionnaire survey was conducted with the parents of children who had an abnormal hearing screening result in 2024/2025. An original questionnaire to follow up on children with abnormal hearing screening was created for the prospective part of the study. Parents of these children were given a QR code linked to an online questionnaire one year after screening. Data from this questionnaire were collected from September 2025 to February 2026. The tool LimeSurvey (LimeSurvey Community Edition, Version 5.6.7) was used to create the online version of this questionnaire. The survey included a maximum of nine multiple-choice questions. It was available in German, English, Bosnian/Serbian/Croatian, Hungarian, Romanian, Turkish, and Arabic (representing the languages with the highest native speaker prevalence in Upper Austria) in order to allow for maximum participation. Translations were conducted by professional medical translators.

## 3. Results

The annual speech, language, and hearing screenings in kindergartens of Upper Austria were performed on 113,053 children between 2018 and 2025. The analysis evaluated the number of participants and the number of abnormal hearing screenings annually. The number of children screened ranged from 14,975 in 2024/2025 (October to February) to 17,101 in 2020/2021. The relative frequency of abnormal hearing screenings varied between 5.89% (2020/2021) and 8.17% (2022/2023) (Table 1).

### 3.1. Detailed Analysis of Screening 2022/2023

In 2022/2023, a total of 15,829 children were screened for speech and hearing in Upper Austria. (Table 1) 12,516 of these screened children were 4 to 5 years old at the time of screening and were therefore included in this study. The remaining 3313 were 5-to-6-year-olds who had missed the screening in the previous year due to absence on the screening day. The mean age of the children was 4.62 (SD 0.39) years. The gender distribution was 6316 (50.99%) male and 6070 (49.01%) female. In total, 9175 (74.02%) of the children were monolingual, and 3220 (25.98%) were multilingual. The prevalence of abnormal hearing screenings was 6.57%. Regarding grammar, the screening was normal in 9855 (81.32%) of the children and abnormal in 2263 (18.68%). In the articulation screening, 3886 (31.72%) children had normal results and 8364 (68.28%) had abnormal results (Table 2). In Addition, an analysis was performed on factors such as gender, mono- or multilingualism, and speech screening results in relation to the results of the hearing screening. Among multilingual children, 3.76% had an abnormal hearing screening, whereas monolingual children had an abnormal hearing screening in 7.55%. In contrast, 28.29% of multilingual children had unclear hearing screening results compared to 9.9% of monolingual children. Similarly, a discrepancy was found in the grammar category.

Only negligible associations were observed between abnormal HS outcomes and speech testing outcomes (grammar: φ = −0.02, *p* = 0.02; articulation: φ = 0.02, *p* = 0.06), with no statistically significant association observed for articulation. In contrast, unclear HS results showed a small positive association with grammar outcomes (φ = 0.28, *p* < 0.001), indicating that unclear HS outcomes were more frequent among multilingual children. Multilingualism was also associated with unclear HS outcomes (φ = −0.23, *p* < 0.001), indicating that unclear HS results were more frequent among multilingual children. The association between unclear hearing screening results and articulation outcomes was weak (φ = 0.09, *p* < 0.001). A weak positive association was also observed between grammar and articulation outcomes (φ = 0.17, *p* < 0.001). Multilingualism showed a moderate association with grammar outcomes (φ = −0.37, *p* < 0.001), indicating more frequent abnormal grammar results among multilingual children, whereas its association with articulation outcomes was negligible (φ = −0.05, *p* < 0.001).

**Figure 1:**
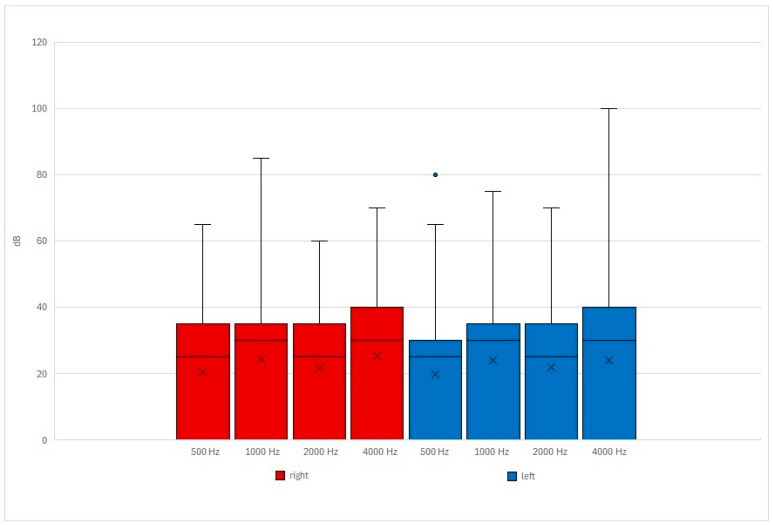
Analyses of the audiogram in children with abnormal hearing screenings. Right and left ear separately at the four principal frequencies.

The audiograms were available for 12,396 children. 9763 (78.76%) audiograms were qualified as normal, 814 (6.57%) as abnormal, and 1819 (14.67%) as unclear. Audiograms of the abnormal hearing screenings (*n* = 814) were analyzed in detail (Table 3, Figure 1). In some cases, no value was recorded for at least one frequency (*n* = missing). The mean PTA4 was 23.2 dB for the right ear and 22.6 dB for the left ear. Of the 814 abnormal screenings, 317 showed only one abnormal hearing threshold, whereas 29 showed an elevated threshold across all frequencies.

### 3.2. Analyses of Survey Data

A total of 1090 written invitations to complete the survey were given out to the parents of children with an abnormal hearing screening result in the previous year (September 2024 to January 2025). All of these parents received a recommendation to see an ORL specialist with their child, directly after they were told about the abnormal hearing screening in the previous year. A total of 209 questionnaires were completed, resulting in a return rate of 19.2%. The majority of parents completed the German version of the questionnaire (91.9%), while fewer chose the Bosnian/Serbian/Croatian (1.9%), Hungarian (1.4%), Romanian (2.4%), Turkish (1.4%), or Arabic (1.0%) versions of the questionnaire.

A total of 146 (69.9%) followed the recommendation to see an ORL specialist, and 45 (21.5%) did not. Roughly half (69 children, 47.6%) of the children, who visited an ORL specialist, had received a diagnosis. Middle ear effusion (45 children) and Eustachian tube dysfunction (28 children) were the most common diagnoses; fewer children were diagnosed with upper airway infections (7 children), cerumen (6 children), or other diagnoses.

**Figure 2:**
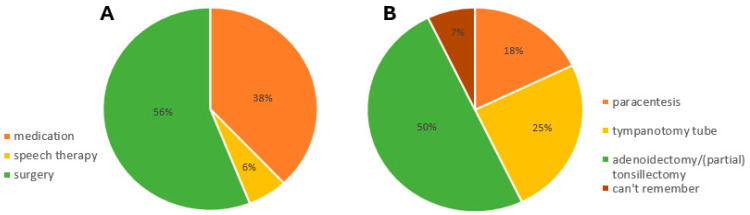
Distribution of treatment after abnormal hearing screening (as stated by parents in the survey questionnaire). (**A**) Distribution of kinds of treatment; (**B**) distribution of kinds of surgery.

Among these children diagnosed with ORL disorders, treatment recommendations were given in 50 cases (Figure 2), and 19 children received no recommendations. Surgery was recommended in 28 cases, medication in 19 (e.g., steroid-containing nasal spray), and speech therapy in 3. Recommended surgical interventions were adenoidectomy and/or (partial) tonsillectomy (14 children), tympanotomy tubes (7 children), and paracentesis (5 children). Moreover, 21 of the recommended interventions had already been performed at the time of the survey, six children were still waiting for their surgical appointment, and one family had decided against surgery.

Of the 45 families who did not act on the recommendation of seeing an otorhinolaryngologist with their child, eight thought it was not necessary, eight sought the advice of another specialist (e.g., pediatrician), five were already seeing an otorhinolaryngologist regularly, and two decided to wait for the problem to resolve spontaneously. When asked for their opinion on the hearing screening in kindergarten, 143 families (68.4%) thought it was very useful. Another 46 (22%) thought it was rather useful, whereas 13 families (6.2%) rated the screening as not very useful, and 4 gave no opinion.

## 4. Discussion

Our retrospective data analysis included data from a total of 113,053 children who participated in the Upper Austrian kindergarten screening between 2018 and 2025, at the age of 4 to 5 years. This large dataset allowed us to obtain a reliable estimate of the current prevalence of potential hearing impairments in preschool-aged children. A comparison of the screening years from 2018 to 2025 reveals a variable number of screened children. This is primarily due to the varying birth rates between cohorts [9]. Furthermore, children who were unable to participate in the scheduled screening year were included in the screening program the following year.

In the presented study group, a slightly higher proportion of abnormal hearing screening findings was observed among male children. These differences were also observed in other studies [13]. Among multilingual children, a lower proportion of abnormal hearing findings was found compared to monolingual children. This may be the result of a more thorough assessment and follow-up in bilingual children, or it may reflect differences in referral patterns and clinical attention. However, a considerably higher number of unclear findings were present among multilingual children, which may relativize this result. The large number of unclear HS outcomes may be attributable to communication difficulties between the examiner and the child due to a language barrier.

It has to be emphasized that the number of unclear HS exceeds the number of abnormal HS outcomes by more than a factor of two. The unclear hearing results are due to incomplete or missing HT measurements that preclude a definitive assessment of the HS. The reasons for incomplete HTs might include reduced attention or cooperation, limited understanding of the instructions, or hearing difficulties themselves. The association between multilingual status and unclear HS outcomes suggests that these unclear screenings are not distributed randomly within the study population. Therefore, they do not represent randomly missing observations, but rather a valid outcome category of an HS and need to be considered as such when interpreting the data.

The positive association between unclear HS outcomes and grammar suggests that the factors that influence grammatical testing also influence the ability to obtain reliable HTs. A conclusion regarding causality cannot be drawn from these associations.

No meaningful associations could be found between either abnormal HS outcomes and grammar or articulation. The weak positive association between grammar and articulation suggests that the factors that contribute to these abnormalities overlap only to a limited extent. It should be noted, however, that the prevalence of abnormal results in the ‘articulation’ category was striking. Nevertheless, since children aged 4 to 5 are still in the developmental phase of proper articulation, this result should be put into perspective [14]. The screening aims for an early detection of all children who might be at risk of developing pathologies of speech sound production. The threshold for an abnormal result in the screening is therefore set relatively low, and many of the children with an abnormal screening result do not have an articulation disorder in the stricter sense. Some of them are referred for further speech diagnostics and therapy, while others can be given time to catch up to speech developmental goals and receive follow-up examinations.

Analysis of the hearing thresholds shows similar distributions between the left and right side across all frequencies. The interquartile ranges are comparable, with a slightly larger spread at 4000 Hz, which indicates increased variability. The medians and means follow a similar frequency-dependent trend for both sides, which suggests no systematic asymmetries.

The highest mean hearing threshold values were found at 4000 Hz. A study by Chow et al. yielded similar results [15]. In this study, a decrease in hearing performance in the high-frequency range (4000–8000 Hz) was observed when examining hearing thresholds in patients with otitis media with effusion. Due to the screening audiogram used in our study, only frequencies up to 4000 Hz were tested, making it impossible to compare at higher frequencies.

The results of the retrospective analysis are comparable to other hearing screening projects in Austria and internationally. Weichbold et al. published a study in which 2199 kindergarten children in Tyrol were examined [16]. Hearing screening was conducted, and children with abnormal hearing results were followed up to determine the cause of hearing loss. In most cases (53%), hearing loss due to middle ear effusion was found, which can also be compared with our data from the prospective part of the study.

Holzinger et al. published a study examining school children with inner ear hearing impairment in Carinthia. This study showed that inner ear hearing loss develops in about half of children only during the first few years of life and is not yet present at the time of the newborn hearing screening [2]. Therefore, hearing screening, as carried out in Upper Austria for children aged 4 to 5 years, is a very important step in the early detection of hearing disorders. Chen et al. conducted a hearing screening of preschool children in China’s Hubei province, examining 28,546 children who had previously undergone a normal newborn hearing screening. This study showed similar results regarding late-onset sensorineural hearing loss in preschool children, with 1.89% of the children exhibiting hearing impairment [17].

Compared to previous studies published in Austria, this investigation includes a larger sample size and encompasses all forms of hearing impairment. The data obtained contribute to creating a comprehensive picture of childhood hearing impairment—not only for Upper Austria but also in the context of other federal states. Comparing the results with findings from other Austrian regions as well as international studies, the importance and necessity of comprehensive hearing screening in the preschool years is clearly demonstrated. Finally, based on this more extensive data, we want to contribute to the further development and standardization of hearing screening programs in all Austrian federal states. Digitization could reduce loss of (written, print) data in the future. The speech sound assessment might be refined so as to reflect the gradation between findings more accurately.

The results of the prospective part of the study have to be interpreted with care, since they are based on the answers that parents gave from memory. Although care was taken to use clear and easily understandable language, we cannot fully exclude the possibility that some parents may not have understood the words completely. Furthermore, some might have had difficulty in correctly recalling details from the otorhinolaryngological examination and/or treatment of their child in the past. Apart from that, the return rate was clearly under the aspired level of 30% and subject to reporting bias. Selection bias cannot be excluded, as families who completed the questionnaire may have had a higher rate of ORL consultations than those who did not complete it or discontinued participation prematurely. This may also be evident in assessments of referral adherence and treatment rates. About 70% of parents were sure they had sought an ORL specialist’s advice, while about 30% had not or could not remember whether they had. Of the children who were examined by an ORL specialist, half received a diagnosis worthy of treatment. Assuming this prevalence for all children with a pathological hearing screening, some parents’ reluctance to visit an ORL specialist would leave about a sixth of the children with a condition that was neither diagnosed nor treated. Yet, about a third of these children were either already in ORL care or taken to another specialty doctor (e.g., pediatrician), who might have diagnosed the underlying condition as well. About 17.8% of parents who did not take their child to an ORL specialist reportedly did not because they did not feel the necessity to do so. It is unclear in the literature how parental concern correlates with identification of children’s hearing disorders. Some studies have shown poor correlation [18]; in others, up to 73% of children with hearing loss were first suspected by their parents [19].

The conditions diagnosed reflect the high prevalence of middle ear effusion and Eustachian tube dysfunction and chronic adenoiditis in the pediatric population [20]. Adenoidectomy with or without concurrent tympanostomy tube placement remains a key role in the treatment of persistent or recurrent OME. In many children, OME can resolve spontaneously; modification of risk factors or execution of auto-insufflation may also be beneficial in selected cases. Current guidelines do not recommend antibiotics, steroids, antihistamines, decongestants, or mucolytics for routine use [21,22]. Several alternative conservative therapies have also been explored [23,24]. Sensorineural hearing loss was not found among these children; although its prevalence doubles between neonatal and preschool age, it is still about 2.2/1000 [2], Because of the low response rate to the follow-up survey of only 19.2%, it is unsurprising that no child with sensorineural hearing loss was identified in a sample of approximately 200 children. A low-response sample does not imply the absence of sensorineural hearing loss in the comprehensively screened population. The results cannot be generalized to the whole screened population regarding the prevalence of delayed-onset sensorineural hearing loss. The majority of children who were referred for surgery had already completed the intervention at the time of the survey, whereas six of them were still waiting for their appointment about one year after they had a pathological hearing screening. Parents rated the screening positively—about 80% of them thought it was very useful or rather useful. These findings suggest that such screening programs increase parental awareness of their children’s hearing problems and motivate them to seek appropriate medical evaluation and treatment that might otherwise not have been pursued. Persistent unidentified or untreated hearing impairment can have long-term adverse consequences for both affected individuals and the healthcare system; however, timely detection through screening offers an opportunity to prevent these outcomes.

## 5. Conclusions

Since children with normal newborn hearing screening results can often develop hearing loss during their first years of life, re-screening children’s hearing at preschool age 4 to 5 is a beneficial preventative measure to ensure the early detection of hearing disorders in early childhood, and enable affected children to receive necessary evaluation and treatment. Left untreated, hearing impairments negatively affect language development and lead to speech delays and significantly poorer school performance. The fact that this study includes a larger sample size than previous investigations, and encompasses all forms of hearing impairment, helps create a more comprehensive picture of childhood hearing impairment. Finally, based on this extensive data, we hope to underscore the importance of the further development and standardization of hearing screening programs in all Austrian federal states, as well as internationally.

## Figures and Tables

**Table 1:** Yearly reports of preschool screening.

Year of Screening (October–February)	No. of Screened Children	No. of Abnormal Hearing Screenings (%)
2018/2019	16,770	1309 (7.81)
2019/2020	16,460	1261 (7.66)
2020/2021	17,101	1008 (5.89)
2021/2022	15,857	1162 (7.33)
2022/2023	15,829	1293 (8.17)
2023/2024	16,061	1157 (7.20)
2024/2025	14,975	1152 (7.69)
Sum	113,053	8342 (7.38)

**Table 2:** Distribution of abnormal and unclear hearing screenings among categories. The percentages are calculated relative to the column total. Missing values were removed from the respective calculations for each variable.

Hearing Screening	Gender			Lingualism			Speech Screening					
							Grammar			Articulation		
	*n* (%)	Female, *n* (%)	Male, *n* (%)	*n* (%)	Mono, *n* (%)	Multi, *n* (%)	*n* (%)	Normal, *n* (%)	Abnormal, *n* (%)	*n* (%)	Normal, *n* (%)	Abnormal, *n* (%)
total	12,386	6070	6316	12,395	9175	3220	12,112	9849	2263	12,250	3886	8364
normal	9760 (78.80)	4866 (80.16)	4894 (77.49)	9762 (78.76)	7574 (82.55)	2188 (67.95)	9631 (79.52)	8262 (83.89)	1369 (60.49)	9706 (79.23)	3274 (84.25)	6432 (76.90)
abnormal	812 (6.56)	391 (6.44)	421 (6.67)	814 (6.57)	693 (7.55)	121 (3.76)	804 (6.64)	679 (6.89)	125 (5.52)	808 (6.60)	232 (5.97)	576 (6.89)
unclear	1814 (14.65)	813 (13.39)	1001 (15.85)	1819 (14.68)	908 (9.90)	911 (28.29)	1677 (13.85)	908 (9.22)	769 (33.98)	1736 (14.17)	380 (9.78)	1356 (16.21)

**Table 3:** Analysis of the audiograms in children with abnormal hearing screenings (*n* = 814).

Right					Left			
	500 Hz	1000 Hz	2000 Hz	4000 Hz	500 Hz	1000 Hz	2000 Hz	4000 Hz
*n* (missing)	13	2	15	18	30	23	30	36
*n* (>30 dB)	292	309	222	331	187	283	234	290
min	0	0	0	0	0	0	0	0
max	65	85	60	70	80	75	70	100
Q25	0	0	0	0	0	0	0	0
Q50	25	30	25	30	25	30	25	30
Q75	35	35	35	40	30	35	35	40
mean	20.37	24.25	21.78	25.41	19.75	24.02	21.88	24.02
sd	17.32	17.62	17.02	18.11	16.66	17.18	17.51	18.54

## Data Availability

The original contributions presented in this study are included in the article. Further inquiries can be directed to the corresponding author.

## Abbreviations

The following abbreviations are used in this manuscript:

ORL	Otorhinolaryngology
ENT	Ears Nose Throat
Hz	Hertz
dB	Decibel
sd	standard deviation
Q25	First quartile (25th percentile)
Q50	Second quartile (50th percentile)
Q75	Third quartile (75th percentile)
e.g.,	exempli gratia
HT	hearing threshold
HS	hearing screening
PTA4	pure tone average at frequencies 0.5, 1, 2, and 4 kHz

## Appendix A

The following table provides details on the preprocessing of the data used in the final evaluation along with the corrective actions taken. In addition to the HT at the four principal frequencies, the questionnaire included an overall assessment of the HS, classified as normal or abnormal. An HT above 30 dB at any frequency should be considered as an abnormal HS. Fields could be marked “999” to indicate that they were intentionally left blank. The questionnaire also allowed for additional remarks to further explain the reason for missing data (“child refused to cooperate, child was uncertain, etc.). Cleaning the data involved resolving contradictions between HS and HT as well as handling missing data and incorrect or inconsistent labels. Additionally, the naming convention for the districts was harmonized. If data was missing for a given parameter (e.g., articulation, gender, etc.), the data was not removed entirely from the analysis, but simply not considered for the evaluation pertaining to that parameter.

**Table A1:** Process of data cleaning.

Step	Issue Type	Data Issue	Action Taken	n
1	Inconsistency/missing data	HS marked “abnormal”, but showed HTs < 30 dB across frequencies.	excluded	6
2	Inconsistency/missing data	HS marked “999”, but showed HTs < 30 dB across frequencies without explanatory remarks.	excluded	112
3	Inconsistency/missing data	HS marked “999”, but showed at least one HT > 30 dB without explanatory remarks.	excluded	2
4	Inconsistency/missing data	HS marked “normal”, but all HTs marked “999”.	HS changed to “999”	14
5	Inconsistency/missing data	HS marked “999”, but all HTs < 30 dB, but with explanatory remark.	Changed all HTs to “999”	175
6	Inconsistency/missing data	HS marked “abnormal”, but all HTs either “999” or <30 dB.	All HTs changed to “999”	132
7	Inconsistency missing data	HS marked “999”, but at least one HT > 30 dB, but with explanatory remark.	All HTs changed to “999”	13
8	Inconsistency/missing data	HS marked “abnormal”, but at least one HT marked “999”.	Changed HS to “999”	4
9	Incorrect labels	Missing values marked as “9”, “99”, or “−1” or left empty instead of “999”.	Changed to “999”	14
10	Incorrect labels	HT marked “40–50” instead of 45.	Changed to 45 dB	1
